# Mapping patterns of abiotic and biotic stress resilience uncovers conservation gaps and breeding potential of *Vigna* wild relatives

**DOI:** 10.1038/s41598-020-58646-8

**Published:** 2020-02-07

**Authors:** Maarten van Zonneveld, Mohamed Rakha, Shin yee Tan, Yu-Yu Chou, Ching-Huan Chang, Jo-Yi Yen, Roland Schafleitner, Ramakrishnan Nair, Ken Naito, Svein Ø. Solberg

**Affiliations:** 1 World Vegetable Center, Headquarters, 60 Yi-Min Liao, Shanhua, Tainan 74151 Taiwan; 2 Horticulture Department, Faculty of Agriculture, University of Kafrelsheikh, Kafr El-Sheikh, 33516 Egypt; 3 Univeristi Malaysia Sabah, Batu 10, 90000 Sandakan, Sabah Malaysia; 4 World Vegetable Center, South Asia, ICRISAT Campus, Patancheru, 502324 Hyderabad, Telangana India; 5 Genetic Resources Center, National Agriculture and Food Science Organization, Tsukuba, Ibaraki Japan; 6 Inland Norway University of Applied Sciences, Faculty of Applied Ecology, Agricultural Sciences and Biotechnology, P.O. Box 400, 2418 Elverum, Norway

**Keywords:** Plant breeding, Plant domestication, Conservation biology

## Abstract

This study provides insights in patterns of distribution of abiotic and biotic stress resilience across *Vigna* gene pools to enhance the use and conservation of these genetic resources for legume breeding. *Vigna* is a pantropical genus with more than 88 taxa including important crops such as *V. radiata* (mung bean) and *V. unguiculata* (cowpea). Our results show that sources of pest and disease resistance occur in at least 75 percent of the *Vigna* taxa, which were part of screening assessments, while sources of abiotic stress resilience occur in less than 30 percent of screened taxa. This difference in levels of resilience suggests that *Vigna* taxa co-evolve with pests and diseases while taxa are more conservative to adapt to climatic changes and salinization. Twenty-two *Vigna* taxa are poorly conserved in genebanks or not at all. This germplasm is not available for legume breeding and requires urgent germplasm collecting before these taxa extirpate on farm and in the wild. *Vigna* taxa, which tolerate heat and drought stress are rare compared with taxa, which escape these stresses because of short growing seasons or with taxa, which tolerate salinity. We recommend prioritizing these rare *Vigna* taxa for conservation and screening for combined abiotic and biotic stress resilience resulting from stacked or multifunctional traits. The high presence of salinity tolerance compared with drought stress tolerance, suggests that *Vigna* taxa are good at developing salt-tolerant traits. *Vigna* taxa are therefore of high value for legume production in areas that will suffer from salinization under global climate change.

## Introduction

Legume crops are an important and cheap source of proteins and micronutrients in human diets^1^. These crops also fix nitrogen because of their symbiosis with *Rhizobium* bacteria, which makes them attractive crops for soil improvement in farming systems^2^. However, increased abiotic stress such as heat, drought, and salinity, and high pressure of diseases and insect pests under climate change decrease yield and quality of existing legume varieties^3^.

To develop varieties that can sustain legume production under climate change, crop wild relatives would be important sources to introgress traits in crop varieties, which help to tolerate, escape, or avoid abiotic stresses and to resist against pests and diseases^4,5^. So far, genetic improvement of legume crops lags behind compared with cereal crops^1^, and legume breeders have underutilized crop wild relatives in developing varieties because of limited information on traits of economic importance in wild species, linkage drag of undesired traits and crossing barriers^6^. A better understanding of trait distribution across legume gene pools will help exploiting legume crop diversity and identifying conservation gaps in legume crop gene pools. In combination with advances in functional genomics, gene editing, and phenotyping, this can broaden the currently narrow genetic basis in legume breeding^1,5,6^.

In this paper, we focus on *Vigna*, to understand patterns of distribution of abiotic and biotic stress resilience across legume gene pools. *Vigna* is a complex and pantropical genus of more than 88 taxa, which are principally diploid and selfing. The genus includes a number of important legume crops for food and nutrition security in tropical Asia and Africa such as *V. radiata* (mung bean) and *V. unguiculata* (cowpea, vegetable cowpea, and yard-long bean) as well as several neglected and underutilized crops such as *V. aconitifolia* (moth bean) and *V. subterranea* (Bambara groundnut).

The optimum temperature range for legume crops is between 10–36 °C and various climatic models predict that temperatures will increase on average with 4 °C by the end of this century^7^. A principal production challenge, which is considered in *Vigna* breeding is therefore heat stress >40 °C^8–10^. Other production challenges relate to drought stress, water logging, and salinity^8–10^, and in the case of cowpea also phosphorus-use inefficiency^8^. *Vigna* wild relatives could contain traits to respond to these abiotic-stress related production challenges.

Traits of breeding interest to escape heat and drought stress include early flowering and maturation. Important morphological and physiological traits in *Vigna* screening for drought tolerance are the number and diameter of xylem vessels, root suberization, stomatal leaf conductance, and low leaf hydraulic conductance, among others^11^. The development of long roots or tubers can help avoiding drought stress but may come at the costs of reduced leaf, pod, or bean productivity^12^. Traits to tolerate heat tolerance include membrane stability, pollen viability, and anther dehiscence, and decline in GABA (γ-aminobutyric acid) declination and increase in antioxidant enzymes, among other traits during plant development^10,13,14^. Traits related to salinity tolerance include relative quantum yield of photosystem II, leaf Na+ and K+ concentrations, malondialdehyde (MDA) levels, and antioxidant enzyme activities^15,16^.

Major production challenges related to biotic stress for several *Vigna* crops include legume pod borer and Yellow Mosaic Disease (YMD) (R. Nair, pers. comm., World Vegetable Center). Specific challenges for *V. radiata* include powdery mildew, *Cercospora* leaf spot; and resistance to the following five pests: bruchids, *Thrips* spp., bean flies (*Ophiomyia spp*. and *Melanagromyza* spp.), and whitefly (*Bemisia tabaci*)^17^. *Vigna unguiculata* is also susceptible to bacterial blight (*Xanthomonas axonopodis* pv *phaseoli*), the parasitic weed striga, viruses such as bean common mosaic virus (BCMV), and anthracnose (*Colletotrichum destructivum*)^8^. There are several traits involved in plant defence against these pests and diseases such as colour, texture, hardness, leaf age, size, spines, trichomes, and chemical constituents in both plants and storage seeds^18,19^.

Owing to the larger number of traits related to abiotic and biotic stress resilience, it is a challenge to carry out a systematic assessment to provide insights in trait distribution and evolution across crop gene pools. In this study, we therefore examine the distribution of traits related to abiotic stress resilience across four *Vigna* gene pools by carrying out a systematic ecogeographic analysis for heat and drought stress. Such an analysis indicates the adaptation of crop wild relatives to hot, dry, or humid growing conditions, independent of a specific trait^20,21^. We develop four *Vigna* gene pools by combining existing information on *Vigna* phylogenetics with information on crossing compatibility. The results of the ecogeographic analysis are compared with reports of systematic screening of *Vigna* taxa for root and shoot biomass accumulation under drought conditions and biomass accumulation under high salinity concentrations coupled to relative quantum yield of photosystem II and leaf Na+ and K+ concentrations^11,16^. Furthermore, we carry out a comprehensive literature review on biotic stress resilience for important pest and diseases, and compare the distribution of these traits with the distribution of traits related to abiotic stress resilience. Finally, we assess the *ex situ* conservation status of *Vign*a to check out germplasm availability for breeders and to target countries for missions to collect germplasm of *Vigna* taxa, which are not well conserved *in situ* and *ex situ*.

## Methods

Four *Vigna* gene pools with 88 taxa from three subgenera and nine biosystematics sections were defined for nine domesticated *Vigna* taxa as listed in GRIN taxonomy^22^. We excluded other taxa because they are part from sections, which are remote to domesticated *Vigna* taxa. We delineated four gene pools following intercrossing studies and complemented with information from genomic, phylogenetic, and biosystematics studies (Text S1). Taxa names were revised according to GRIN taxonomy^22^ and Iseki *et al*.^16^ (Table S1). The *Vigna ex situ* conservation status was determined with data from the 2017 WIEWS database^23^ and the online platform Genesys^24^.

### Collection of presence records

In total, 28,313 georeferenced presence records for 84 of the 88 *Vigna* taxa were used for the detection of geographic patterns of taxonomic richness and gaps in genebank collections. Four *Vigna* taxa did not have any record reported. The presence records come from four data sources:

1. Presence records from herbaria, which were reported by Tomooka *et al*.^25^ were manually curated and where possible georeferenced in Google Earth or with support of www.geonames.org.
2. Presence records from herbaria and living collections, which were stored in the Global Biodiversity Information Facility (https://www.gbif.org/) were collected with the rgbif package^26^ and manually georeferenced if taxa had less than 30 georeferenced presence records.
3. Presence records from genebanks listed in the 2017 WIEWS database^23^.
4. Presence records from the *Vigna* genebank collection of the World Vegetable Center (WorldVeg) were manually curated and where possible georeferenced in Google Earth or with support of www.geonames.org.

### Cleaning of presence records

Presence records with inconsistencies between countries as reported in the passport data and at the projected locations outside a border buffer zone of 10 arc minutes were removed following van Zonneveld *et al*.^27^. Coordinates of presence records located in coastal waters within a 10-arc minute buffer zone to the coastline were relocated to the nearest point in the coastline. Presence records with coordinates from country centroids were removed because these points are likely georeferenced at country level with low precision. For each taxon, duplicate records with the same coordinates were removed to reduce sample bias.

Outlier presence records with climate values far beyond taxa’ niche margins were removed from our dataset because these are likely errors in coordinates or taxonomy. We removed outliers when the values of three or more of 19 bioclimatic variables were outside a threshold of 2.5 times the interquartile range below the first quartile or above the third quartile following van Zonneveld *et al*.^27^. Climate data were derived from the 2.5-arc minute environmental layers of the 2.0 WorldClim database^28^.

### Gap analysis

We identified taxonomic and geographic gaps in genebank collections in two steps. First, to target germplasm from taxa and countries underrepresented in *ex situ* collections, we compared sampled taxonomic richness reported by genebanks and living collections with sampled taxonomic richness reported by herbaria. Second, to detect geographic areas where taxa have not been reported before, we applied ecological niche modelling with Maxent, a widely used modelling algorithm to detect areas where climate conditions are suitable for plant species^29^. We modelled the distribution of each *Vigna* taxon under current climate conditions (1970–2000) using a selection of seven bioclimatic variables available from the WorldClim 2.0 database^28^ (Text S2): These bioclimatic variables were selected after the removal of correlated variables with a Pearson correlation coefficient of more than 0.7. We used the threshold value of maximum specificity + sensitivity to differentiate suitable from not-suitable areas for taxa presence^30^. To reduce sampling bias, we first took the average Maxent results for each taxa from three runs each time, using 80% of the randomly resampled records from grid cells with a size corresponding to 10% of the longest inter-point distance following Fourcade *et al*.^31^. Second, to allow Maxent to discriminate areas with presence records from the areas with no data, we randomly extracted ten times more background points from the area enclosed by the convex hull polygon based on the presence records, and extended with a buffer corresponding to 10% of the longest inter-point distance following van Zonneveld *et al.*^27^. Third, to reduce the risk of including modelled areas where the taxa do not occur in reality, we limited the modelled distribution range by the area enclosed by the convex hull polygon based on the presence records and extended with the buffer around it following van Zonneveld *et al*.^27^.

### Assessment of abiotic stress and biotic stress resilience

We did a literature review to assess biotic stress resistance of *Vigna* taxa with a focus on bruchids (*Callosobruchus* spp.) and Yellow Mosaic Disease (YMD), both of which are important across all *Vigna*, and other pests and diseases as indicated in the introduction. For the assessment of abiotic stress resilience, we carried out an ecogeographic analysis. This analysis was complemented with scores on a 1–5 Likert scale of high to low tolerance of *Vigna* taxa to hydratation and salinity on the basis of biomass accumulation during early growth stages under drought and saline conditions in pot experiments^11,16^. These experiments were carried out by the Genetic Resources Center of the Japanese Agriculture and Food Science Organization. All taxa, which scored a “1” for at least one accession in the pot experiments, were identified in this study as taxa with a high level of tolerance. Taxa, which scored a “2” for at least one accession, were identified as taxa with an intermediate level of tolerance. Taxa with poorer scores were identified as taxa with a low level of tolerance.

The ecogeographic analysis consisted of one-way ANOVAs with ranked climate data to identify *Vigna* taxa, which occur in harsh climate conditions, and which therefore would have acquired traits related to abiotic stress resilience. Climate data was extracted from the WorldClim 2.0 database^28^. We identified four types of harsh climate conditions:

- *Permanently hot* climate conditions with high annual mean temperature;
- *Seasonally hot* climate conditions with high mean temperatures in the wettest quarter;
- *Permanently dry* climate conditions with low annual rainfall; and
- *Seasonally dry* climate conditions with low rainfall in the wetter quarter.

We selected temperature and rainfall in the wettest quarter as indicators for *seasonally hot* and *seasonally dry* climate conditions because these are critical variables for flowering and seed development of annual plants such as *Vigna* taxa (pers. comm., Ken Naito, Genetic Resources Center, Japanese Agriculture and Food Science Organization). Post-hoc Tukey Honest Significant Difference (HSD) tests were carried out to identify *Vigna* taxa, which occur in harsh climate conditions; these taxa belonged to the HSD group with highest temperature or lowest rainfall values. We then identified a second group of *Vigna* taxa, which occur in less harsh climate conditions; these taxa belonged to the HSD group with second-highest temperature or second-lowest rainfall values. Finally, all other *Vigna* taxa were assigned to a third group because they occur in less harsh climate conditions. For nine taxa, less than five georeferenced records were available to extract climate data. Because of this lack of sufficient data, these taxa were excluded from the ecogeographic analysis.

### Software

We performed all analyses in R version 3.3.3. Taxonomic richness maps were developed with R version 3.3.3 and the ggplot2 version 3.1.0^32^, and with the use of a freely available global political map^33^. The code and datasets are available at https://figshare.com/articles/R_Script_and_dataset_of_Vigna_ecogeographic_analysis/7551011. Text S3 lists all R packages, which were used in this study.

**Figure 1:**
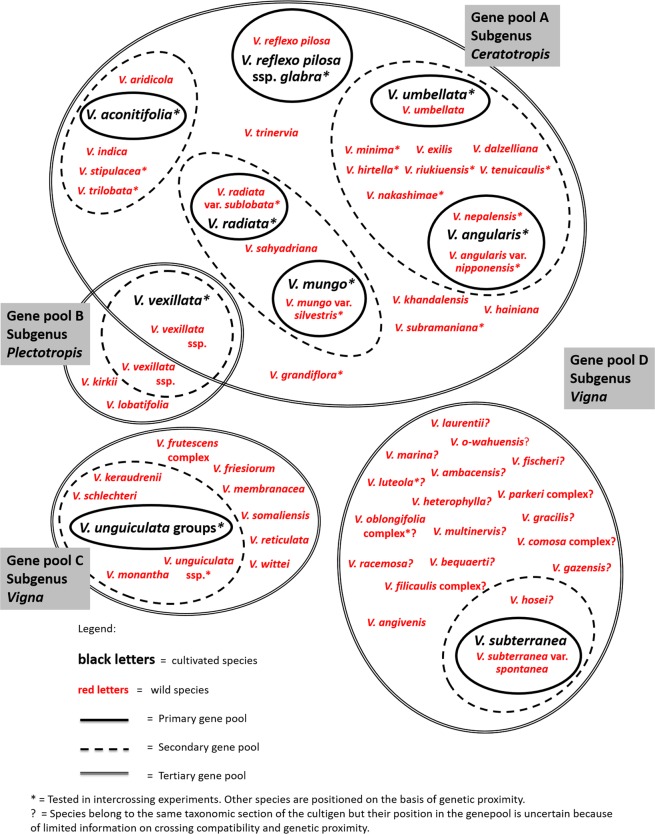
*Vigna* gene pools.

## Results

### Vigna gene pools

Gene pool A consists of Asian *Vigna* taxa of the subgenus *Ceratotropis*, including *V. radiata* (mung bean), *V. mungo* (urd bean), *V. angularis* (azuki bean), *V. umbellata* (rice bean), *V. reflexo-pilosa* var. *glabra* (creole bean), and *V. aconitifolia* (moth bean) (Fig. 1). *Vigna radiata* is the economically most important *Vigna* of gene pool A. *Vigna radiata* can produce mature seeds within two months, which makes it popular for crop diversification of rice and wheat systems^34^. The domestication centre of *V. radiata* is thought to be in current India. *Vigna mungo* is also thought to be domesticated in current India and is genetically close to mung bean, and is especially popular in southern India and Pakistan. *Vigna angularis* is mainly cultivated in Japan and Korea, and is thought to be domesticated in Japan^35^. Other domesticated taxa are minor crops with important features. *Vigna umbellata* can grow on poor lands and is mostly cultivated in the tropical Asian highlands^36^. *Vigna reflexo-pilosa* var. *glabra*, which is the only known tetraploid *Vigna* taxa^25^, is cultivated in Viet Nam, the Philippines, Mauritius, and Tanzania^25^. *Vigna aconitifolia*, another legume crop is thought to be domesticated in current India is cultivated where it is too hot for mung bean cultivation^36^. In addition, several wild taxa are locally consumed and cultivated in Southeast Asia including *V trilobata* (jungle bean) and *Vigna trinervia* (tooapee)^37,38^.

**Figure 2:**
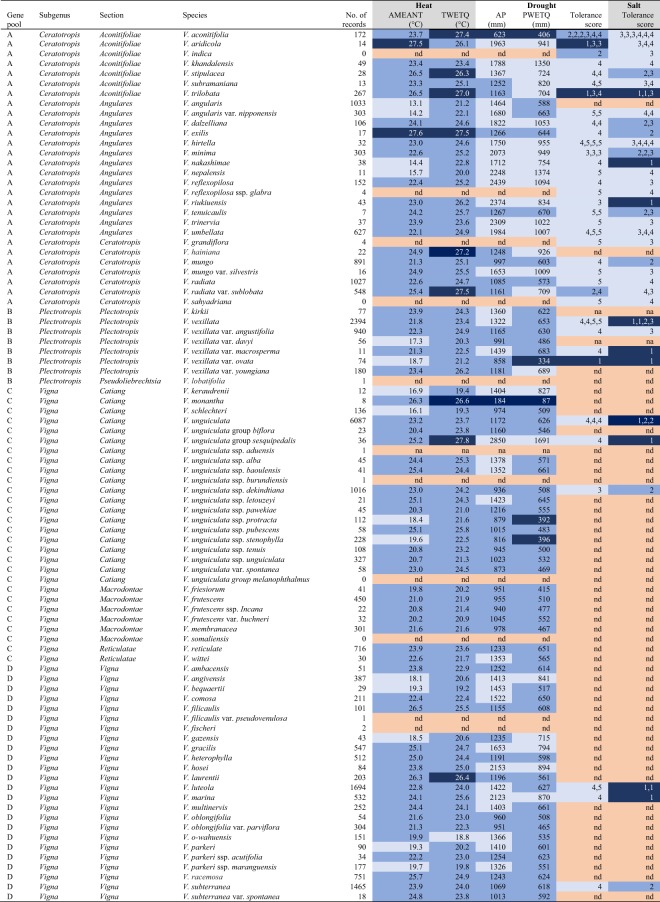
Abiotic stress tolerance of 88 *Vigna* species. The tolerance of these species was assessed for six variables: (1) Mean value of Annual Mean Temperature across the distribution of presence records (AMEANT); (2) Mean value of Temperature in the Wettest Quarter (TWEQ); (3) Mean value of Annual Precipitation (AP); (4) Mean value of Precipitation in the Wettest Quarter (PWETQ); (5) Drought tolerance score after lab screening carried out by Iseki *et al*.^11^ in which 1 indicates high tolerance while 5 indicates low tolerance; (6) Salinity tolerance score after lab screening carried out by Iseki *et al*.^16^ in which 1 indicates high tolerance while 5 indicates low tolerance. The four climate variables were calculated with data from WorldClim^28^. The colour of the cells indicates the level of tolerance for a specific variable. **Darkblue** indicates presence in harsh climate conditions or a high tolerance score in pot experiments. **Intermediate blue** indicates presence in intermediate climates or a suboptimal tolerance score in pot experiments. **Light blue** indicates presence in temperate or humid climate conditions or a poor tolerance score in pot experiments. **Salmon** indicates no data (nd).

Gene pool B comprises taxa from subgenus *Plectotropis* to which the domesticated *V. vexillata* (tuber cowpea) and its wild relatives belong (Fig. 1). There are two types of domesticated *V. vexillata*. The tuber type is domesticated in Asia while the pea type was domesticated in Africa^39^.

Gene pool C comprises taxa of the sections *Catiang*, *Macrodontae*, and *Reticulatae* of subgenus *Vigna* (Fig. 1). The domesticated taxa in this gene pool are *V. unguiculata* group *sesquipedalis* (yard-long bean), which is mostly cultivated in Asia, and *V. unguiculata* group *unguiculata* (grain and vegetable cowpea), which was domesticated in West and Central Africa. *Vigna unguiculata* group *sesquipedalis* (yard-long bean) distinguishes itself from the *V. unguiculata* group *unguiculata* by its long pod (~1 m). People harvest and eat young pods of *V. unguiculata* group *sesquipedalis*, rather than the beans.

Finally, gene pool D comprises the domesticated *V. subterranea* (Bambara groundnut) and other taxa of section *Vigna* of subgenus *Vigna* (Fig. 1). *Vigna subterranea* is thought to be domesticated in West Africa^40^. Farmers cultivate several wild taxa from this gene pool for local consumption, such as *V. marina* and *V. luteola*^41,42^. Several taxa from this genus are grown widely in the tropics as forages including *V. luteola*, *V. hosei*, and *V. parkeri*^42^. This pantropical section includes taxa from Africa such as Bambara groundnut, from the Americas such as *V. luteola*, and one taxa endemic to Hawaii in the Pacific: *V. o-wahuensis*. Gene pool D is the least investigated of all four gene pools. Many taxa of this gene pool have not yet been included in crossing compatibility experiments.

### Abiotic stress resilience

Nine taxa from three gene pools occur in *seasonally hot* climate conditions and five taxa from three gene pools occur in *seasonally dry* climate conditions (Tables 1, 2). In contrast, only two taxa from just one gene pool occur in *permanently hot* climate conditions and two other taxa from two gene pools occur in *permanently dry* climate conditions. Six taxa from three gene pools tolerated high levels of salinity in contrast to only three taxa from two gene pools, which tolerated high levels of dehydration.

**Table 1:** Patterns of abiotic and biotic stress resilience across the *Vigna* genus.

Main region of distribution	Sections and gene pools	Total no. of taxa	Presence in extreme climates compared with other taxa^a^					Abiotic stress tolerance^b^			Biotic stress resistance^c^			
			No. of taxa evaluated	High AMEANT^d^	High TWETQ^e^	Low AP^f^	Low PWETQ^g^	No. of taxa screened	Dehydration	Salinity	No. of taxa reviewed	YMD^h^	No. of taxa reviewed	Bruchids
Asia	Section *Aconitifoliae*	7	6	1	3	1	1	7	1	0	2	2	3	1
Asia	Section *Angulares*	14	13	1	1	0	0	13	1	3	4	4	11	9
Asia	Section *Ceratotropis*	7	5	0	2	0	0	6	0	0	3	3	5	3
Asia	Total gene pool A	28	24	2	6	1	1	26	2	3	9	9	19	13
Africa	Section *Plectotropis*	7	7	0	0	0	1	4	1	3	0	na	1	1
Africa	Section *Pseudoliebrechtsia*	1	0	na	na	na	na	0	na	na	0	na	0	na
Africa	Total gene pool B	8	7	0	0	0	1	4	1	3	0	na	1	1
Africa	Section *Catiang*	20	17	0	2	1	3	3	0	2	1	1	1	1
Africa	Section *Macrodontae*	6	5	0	0	0	0	0	na	na	0	na	0	na
Africa	Section *Reticulatae*	2	2	0	0	0	0	0	na	na	0	na	1	1
Africa	Total gene pool C	28	24	0	2	1	3	3	0	2	1	1	2	2
Africa	Section *Vigna*	24	22	0	1	0	0	3	0	2	0	na	2	2
Africa	Total gene pool D	24	22	0	1	0	0	3	0	2	0	na	2	2

^a^Data analysis obtained in the ecogeographic analysis of this study; ^b^Screening for drought and salinity tolerance carried out by Iseki *et al*.^11,16^; ^c^Literature review carried out in this study; ^d^AMEANT: Annual Mean Temperature; ^e^TWETQ: Temperature in the Wettest Quarter; ^f^AP: Annual Precipitation; ^g^PWETQ: Precipitation in the Wettest Quarter; ^h^YMD: Yellow Mosaic Disease; na: not applicable.

**Table 2:** Percentages of taxa, sections, and gene pools of *Vigna*, which harbor traits related to abiotic or biotic stress resilience.

Type of Evolutionary Significant Unit (ESU)	Taxa	Sections	Gene pools
No. of ESUs evaluated in ecogeographic analysis	77	8	4
% of ESUs in climates with high annual mean temperature	3%	25%	25%
% of ESUs in climates with high temperature in the wettest quarter	12%	58%	75%
% of ESUs in climates with low annual precipitation	3%	25%	50%
% of ESUs in climates with low precipitation in the wettest quarter	6%	38%	75%
No. of ESUs screened for abiotic stress tolerance	36	6	4
% of ESUs with reported dehydration tolerance	8%	50%	50%
% of ESUs with reported salinity tolerance	27%	67%	100%
No. of ESUs reviewed for resistance against Yellow Mosaic Disease (YMD)	10	4	2
% of ESUs with reported YMD resistance	100%	100%	100%
No. of ESUs reviewed for bruchid resistance	24	7	4
% of ESUs with reported bruchid resistance	75%	100%	100%

The wild *V. trilobata*, *V. vexillata* var. *ovata*, and *V. monantha* and the domesticated *V. aconitifolia* returned highest scores for abiotic stress resilience (Fig. 2). These four taxa showed high levels of abiotic stress resilience for three of the six variables. *Vigna trilobata* occurs in *seasonally hot* climate conditions and tolerated high levels of dehydration and salinity. *Vigna vexillata* var. *ovata* occurs in *seasonally dry* climate conditions and tolerated high levels of dehydration and salinity. *Vigna monantha* and *V. aconitifolia* occur in *permanently dry* and *seasonally hot* climate conditions with low rainfall during the wettest quarter. While *V. monantha* was not screened for dehydration or salinity tolerance, *V. aconitifolia* accessions did not tolerate high levels of dehydration or salinity.

**Figure 3:**
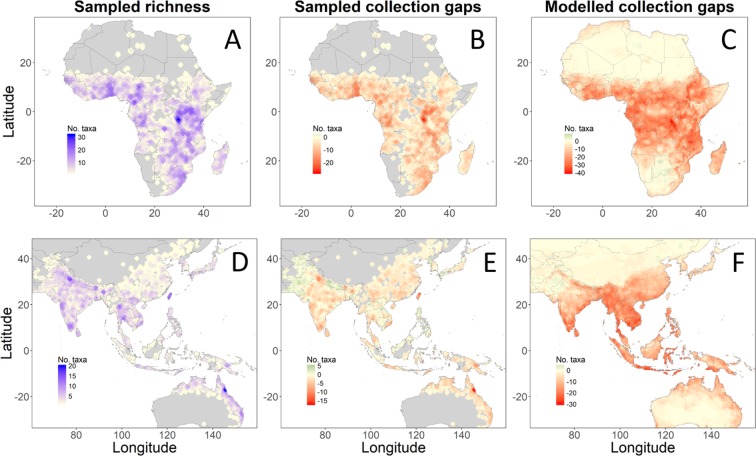
Sampled taxonomic richness and sampled and modelled collection gaps. Panel A and D show sampled taxonomic richness; Panel B and E show sampled taxonomic richness, which is not conserved *ex situ*; Panel C and F show gaps where a high number of taxa are modelled to occur but are not reported in herbaria, living collections, and genebanks. Maps were made by Maarten van Zonneveld in R version 3.3.3 with ggplot2 version 3.1.0.

The wild *V. aridicola*, *V. exilis, V. laurentii* and the domesticated *V. unguiculata* group *sesquipedalis* showed high levels of abiotic stress resilience for two of the six variables (Fig. 2). *Vigna aridicola* occurs in *permanently hot* climate conditions and tolerated high levels of dehydration. *Vigna exilis* occurs in *permanently* and *seasonally hot* climate conditions. This species, however, did not tolerate high levels of dehydration or salinity. *Vigna laurentii* occurs *in seasonally hot* and *seasonally dry* climate conditions. This species was not tested for dehydration and salinity tolerance. Finally, *V. unguiculata* group *sesquipedalis* occurs in *seasonally hot* climate conditions and tolerated high levels of salinity.

Thirteen taxa showed high levels of abiotic stress resilience for one of the six variables. *Vigna hainiana*, *V. radiata* var. *sublobata*, and *V. stipulacea* occur in *seasonally hot* climate conditions. *Vigna heterophylla*, *V. kirkii*, and *V. unguiculata* subsp. *stenophylla*, occur in *seasonally dry* climate conditions. Finally, high levels of salinity tolerance were reported for the domesticated *V. unguiculata* and *V. vexillata*, and the wild *V. luteola*, *V. marina, V. nakashimae*, *V. riukiuensis*, and *V. vexillata* var. *macrosperma*.

### Biotic stress resistance

Bruchid resistance was reported in 18 of the 24 taxa (75%), which were evaluated in literature. These 18 taxa included representatives from all four gene pools (Table 2; S2). However, no resistance against bruchids was reported for mung bean. YMD resistance was reported in all ten taxa (100%), which were evaluated, and which belonged to gene pool A of *V. radiata* and other Asian *Vigna* crops and gene pool C of *V. unguiculata* group *unguiculata* and *unguiculata* group *sesquipedalis*.

For the other pest and diseases, remarkably less research has been conducted. In gene pool A, only for germplasm of *V. radiata* and *V. mungo*, resistance was reported against legume pod borer, whiteflies, stem borer and *Thrips* spp. In gene pool A, no reports were found for resistance against powdery mildew, bacterial blight, or *Cercospora* leaf spot. In gene pool C, germplasm of the primary gene pool of *V. unguiculata* group *unguiculata* and *unguiculata* group *sesquipedalis* was reported to resist against bacterial blight, *Thrips* spp., and legume pod borer. No reports were found on resistance against anthracnose. Little research was done on biotic stress resistance in gene pools B and D, except for bruchid resistance and cowpea mottle carmovirus (CPMoV).

### *Ex situ* conservation status

In 2017, 96 institutions conserved *ex situ* 89,288 accessions of the targeted *Vigna* taxa. Eight institutes maintained more than 53,756 of these accessions (60%) (Table S3). As a safety duplicate, in 2018, 31,500 accessions from 25 taxa were stored in the Svalbard Global Seed Vault (SGSV, 2018, https://www.nordgen.org/sgsv/), mainly from the International Institute of Tropical Agriculture (IITA), the Centro Internacional de Agricultura Tropical (CIAT), and WorldVeg. From the domesticated taxa, IITA held the largest collections of *V. unguiculata* (cowpea) and *V. subterranea* (Bambara groundnut). The status of *V. unguiculata* group *sesquipedalis* (yard-long bean) collection is unclear because not all genebanks provided taxonomic data below species level, which is necessary for this crop. WorldVeg held the largest collection of *V. radiata* (mung bean) and *V. angularis* (azuki bean). The Indian Bureau of Plant Genetic Resources (NBPGR) held the largest collections of *V. mungo* (urd bean), *V. umbellata* (rice bean), and *V. aconitifolia* (moth bean). CIAT held the largest collection of *V. vexillata* (tuber cowpea). The Genetic Resources Center of the Japanese Agriculture and Food Science Organization held the largest collection of *V. reflexo-pilosa* (creole bean). The Australian Grains genebank and IITA maintained important collections of African wild *Vigna* while the Japanese Agriculture and Food Science Organization and NBPGR held an important collection of wild Asian *Vigna*. The national botanic garden of Belgium, Meise held the most diverse *Vigna* collection but only keeps a limited number of accessions per taxon.

### Collection gaps

Two Asian *Vigna* and four African *Vigna* were not represented in any of the genebanks, which reported to WIEWS: *Vigna sahyadriana, V. indica*, *V. keraudrenii*, *V. monantha*, *V. somaliensis*, and *V. gazensis*. Genebanks maintained less than 10 accessions for nine other Asian *Vigna* and seven other African *Vigna* (Table S3). Priority countries for germplasm collecting missions of these 22 poorly- conserved taxa in Asia are Thailand, India, Sri Lanka, and Myanmar (Table S4). In Africa, priority countries are Madagascar, DRC Congo, South Africa, Benin, Burundi, Somalia, Namibia, and Tanzania. In the Pacific, *V. o-wahuensis* requires urgent collection in Hawaii.

Geographic gaps in Asia and the Pacific with high taxonomic richness reported by herbaria but low coverage reported by genebanks and living collections are Taiwan, Northeast Australia, and India (Fig. 3). When comparing the reported overall taxonomic richness with the modelled taxonomic richness, our analysis showed modelled gaps in West Cambodia, Central Thailand, South Viet Nam, and coastal India (Fig. 3). Geographic gaps in Africa with high reported taxonomic richness but low coverage by genebank collections are Burundi, Benin, and Uganda. In addition, East DRC Congo is a collection gap following the modelled richness analysis.

## Discussion

### Genus-wide screening of *Vigna* reveals an evolutionary pattern of high biotic vs. low abiotic stress resilience

Our findings show that sources of pest and disease resistance occur in at least 75 percent of the taxa, which were part of screening assessments. In contrast, sources of abiotic stress resilience occur in less than 30 percent of the taxa, which were part of screening assessments. We therefore hypothesize that during evolution, *Vigna* taxa have been conservative in acquiring traits related to abiotic stress resilience compared with pest and diseases resistance. To verify the pattern of high biotic vs. low abiotic stress resilience for the whole *Vigna* it is necessary to screen more *Vigna* taxa from gene pools C and D, which belong both to *Vigna* subgenus *Vigna* because these are under-represented in the screening studies, which were reported in this study.

 Fifteen percent of the taxa screened in the ecogeographic analysis occur in *seasonally hot* or *seasonally dry* climate conditions, where it is possible to escape heat and drought stress through rapid flowering and maturation rather than to tolerate heat and drought stress. In contrast, five percent of the taxa occurs in *permanently hot* or *permanently dry* climate conditions, and requires traits to tolerate continuous heat or drought stress. This finding suggests that during evolution *Vigna* taxa more easily acquire phenological traits for short life cycles to escape drought and heat stresses compared with acquiring physiological traits to tolerate these stresses continuously.

Co-evolution can explain the high percentage of taxa, which possesses biotic stress resistance compared with heat and drought stress resilience. Resistance against pest and diseases has often been a result from continuous and recent co-evolution in the geographic areas of occurrence and pressure of pest and diseases^43^. Pest pressure can change genotype frequencies in a plant population within just a few generations^44,45^. For example for wild tomato relatives, the density of trichomes and levels of acylsugar concentrations, related to direct pest defence, correspond with pest pressure and the geographic distribution of these pests^46,47^.

The presence of salinity tolerance in 27 percent of taxa compared with eight percent drought-tolerant taxa in the pot experiments, suggest that *Vigna* taxa are good at developing salt-tolerant traits compared with drought-tolerant traits^16^. Several wild *Vigna* taxa occur naturally in coastal areas and are adapted to a saline environment^41^. These *Vigna* genetic resources are of high value for legume production in geographic areas, which suffer from salinization.

Our findings suggest that the section *Aconitifolia* contains high levels of heat and drought stress resilience. This section includes *V. aconitifolia* (moth bean), which occurs in *permanently dry* and *seasonally hot* climate conditions. This finding contrasts with the low dehydration tolerance of *V. aconitifolia*, which was reported in the pot experiments^11^. Two possible reasons can explain this discrepancy. First, the pot experiment measured dehydration tolerance at an early growth stage while plants can have traits to tolerate or escape drought stress during all growth stages. The ecogeographic analysis captures this broad range of traits with presence records of populations of *V. aconitifolia* in harsh environments. Second, the limited number of accessions of *V. aconitifolia* in the pot experiments may not reflect the full intra-specific genetic variation of this species.

Vice versa, *V. aridicola* from section *Aconitifoliae* tolerated dehydration while the ecogeographic analysis indicated that this species occurs in humid regions. It could be that our analysis does not reflect the complete climatic ranges because we only were able to collect 14 presence records of this species.

Numerous *Vigna* taxa from all four gene pools occur in marginal conditions, on poor sandy soils, or geographic areas that are regularly inundated^41, 42^. We therefore anticipate that many *Vigna* taxa, which have not been phenotyped for abiotic stress resilience yet, have traits related to abiotic stress resilience. The biggest phenotyping gap is in the *Vigna* taxa of gene pools C and D, which belong both to *Vigna* subgenus *Vigna*.

To test if the pattern of high biotic vs. low abiotic stress resilience is common in gene pools of other legume crops, more screening studies are required that simultaneously assess abiotic or biotic stress resilience. We only found one study, which simultaneously screened legume wild relatives for abiotic stress and biotic stress resilience^48^. This study with eight chickpea wild relatives (*Cicer* spp.) focuses on cold stress rather than heat stress in combination with biotic stress resistance. Nevertheless, the study’s results are in line with our findings of high biotic vs. low abiotic stress resilience because the researchers identified four or more *Cicer* taxa resisting bruchids and other pests and diseases compared with only two wild *Cicer* taxa tolerating cold stress.

### Identification of multifunctional traits

Because of the high frequency of taxa with traits related to biotic stress resilience, we expect that taxa with traits related to abiotic stress resilience have also a high chance to include traits related to biotic stress resilience. Our analysis suggests that many taxa from the section *Aconitifoliae* show high levels of abiotic stress resilience, but only a limited number of accessions from this section were screened for resistance against pests and diseases. These and other *Vigna* taxa with high levels of abiotic stress resilience would require further evaluation under different abiotic and biotic stress combinations.

To date, screening and understanding of the ability of *Vigna* taxa to adapt against combined biotic and abiotic stresses is largely unknown. Taxa, which are adapted to hot, drought, or extreme-rainfall conditions could have stacked different traits over time to cope with both abiotic and biotic stresses. In addition, multifunctional traits, such as high levels of antioxidant capacity, could help these taxa to tolerate abiotic and biotic stresses at the same time^49^. In fact, exaptation, which is the evolutionary process of changes in trait functions^50^, predicts that a trait, which initially responded to a single stress, is likely to generate several functions over evolutionary time to eventually respond to multiple stresses.

We propose trichomes as a promising multifunctional trait in *Vigna* taxa for further screening because trichomes help plants to cope with both abiotic and biotic stresses^51^. High density of trichomes on the pods of *V. radiata* and the *V. unguiculata* complex complicates the mobility of the adult of bruchids and pod borers over the pods and decrease pest infestations^18,52^. Numerous studies show that glandular trichomes in tomato wild relatives produce secondary metabolites including acylsugars, methylketones, and sesquiterpenes, which intoxicate, repel, or trap pests^19,53^. At the same time, trichomes may increase abiotic stress tolerance by reducing leaf radiation absorbance and facilitate condensation of air moisture onto the plant surface, among other functions^54^. Further research will reveal the relationships between trichomes types and densities, trichrome evolution, and abiotic and biotic stress resilience in *Vigna* taxa.

### Cross-compatibility between Vigna taxa is poorly understood

For many *Vigna* taxa, genetic relationships and crossing compatibility are still poorly understood. This is especially true for the pantropical *Vigna* section of subgenus *Vigna*, which includes taxa from Africa, the Americas, and the Pacific. Domestication and origin of *Vigna* taxa is largely unknown. For example, where did the *Vigna* genus originate and how did the genus spread across Africa, Asia, the Americas, and the Pacific?

Only few studies reported on biotic stress resilience of wild relatives of *V. unguiculata* (cowpea) compared with wild relatives of *V. radiata* (mung bean). It could be that cowpea breeders do not yet need to use wild relatives of the *V. unguiculata* complex for biotic stress resilience because the many botanical varieties in the primary gene pool of the *V. unguiculata* complex provide sufficient variation for finding traits. Another possibility could be that *V. unguiculata* is difficult to cross with close relatives compared with *V. radiata* and its close relatives, which requires further crossing studies.

### Quarter of Vigna taxa requires urgent conservation actions

Twenty-six percent of the 88 *Vigna* taxa, which were considered in this study, requires urgent germplasm collecting efforts because they are not- or under-represented in genebanks. Two Asian *Vigna* taxa and four African *Vigna* taxa require urgent efforts of germplasm collecting because no genebank had reported the maintenance of these taxa. Nine other Asian *Vigna* taxa and seven other African *Vigna* taxa also require urgent efforts of germplasm collecting because genebanks reported less than 10 accessions of these taxa. Targeted Asian and Pacific countries for germplasm collecting efforts include India, Thailand, Myanmar, Sri Lanka, Australia, and Taiwan. In Africa, our sampled and modelled richness analyses indicate Burundi, Benin, DRC Congo, and Madagascar as priority countries for *Vigna* germplasm collecting efforts..

*Vigna* taxa with high levels of heat and drought stress tolerance are rare. We therefore propose to use the presence of these type of traits as a criterion to prioritize taxa for conservation. The section *Aconitifoliae* requires urgent conservation efforts when considering this criterion in combination with poor genebank coverage. Five out of the seven *Aconitifoliae* taxa are poorly conserved *ex situ* with 10 or fewer reported accessions in genebanks while this section includes several traits related to abiotic stress tolerance. Taxa from this section mainly occur in India and Sri Lanka, which are priority countries for germplasm collecting. In addition to the taxa in this section, *Vigna exilis*, which occurs in *permanently hot* climate conditions and V*. monantha*, which occurs in *permanently dry* conditions, also require urgent conservation because these taxa were not reported in genebanks, except for one accession of *V. exilis*.

The pacific *V. o-wahuensis*, endemic to Hawaii, is critically endangered according to the U.S. Endangered Species Act^56^. Fortunately, 27 accessions have been safeguarded in the Lyon Arboretum, Hawaiian Rare Plant Program (pers. comm. Marian Chau, Lyon Arboretum). Strengthening collaboration between genebanks and botanical gardens such as the Lyon Arboretum and the National Botanic Garden of Belgium in Meise, would further enhance *ex situ* conservation and germplasm availability of *Vigna*.

*Vigna monantha* and another African *Vigna*, *V. keraudrenii* require urgent *in situ* and *ex situ* conservation efforts because these taxa are endangered according to the IUCN Red List^55^. Until now, the IUCN has evaluated only six *Vigna* taxa as part of the Red List. The inclusion of more *Vigna* taxa on the IUCN Red List will help to understand their *in situ* conservation status better.

## Supplementary information

Supplementary Information .
