# Supplementary material for: Mapping patterns of abiotic and biotic stress resilience uncovers conservation gaps and breeding potential of Vigna wild relatives
Source: Sci Rep. 2020 Feb 7;10:2111. doi: 10.1038/s41598-020-58646-8 (PMC7005857; doi:10.1038/s41598-020-58646-8)
Supplement: Supplementary file 1 — Supplementary Information . [file 41598_2020_58646_MOESM1_ESM.docx]

**Supplementary Information for**

**Mapping patterns of abiotic and biotic stress resilience uncovers conservation gaps and breeding potential of *Vigna* wild relatives**

Maarten van Zonneveld^1*^, Mohamed Rakha^1,2^, Shin yee Tan^1,3^, Yu-Yu Chou^1^, Ching-Huan Chang^1^, Jo-Yi Yen^1^, Roland Schafleitner^1^, Ramakrishnan Nair^4^, Ken Naito^5^, Svein Ø. Solberg^1,6^

^1^ World Vegetable Center, Headquarters, 60 Yi-Min Liao, Shanhua, Tainan 74151, Taiwan

^2^ Horticulture Department, Faculty of Agriculture, University of Kafrelsheikh, Kafr El-Sheikh 33516, Egypt

^3^ Univeristi Malaysia Sabah, Batu 10, 90000, Sandakan, Sabah, Malaysia.

^4^ World Vegetable Center, South Asia, ICRISAT Campus, Patancheru 502324 Hyderabad, Telangana, India

^5^ Genetic Resources Center, National Agriculture and Food Science Organization, Tsukuba, Ibaraki, Japan

^6^ Inland Norway University of Applied Sciences, Faculty of Applied Ecology, Agricultural Sciences and Biotechnology, P.O. Box 400, 2418 Elverum, Norway

* [maarten.vanzonneveld@worldveg.org](mailto:maarten.vanzonneveld@worldveg.org)

**This document includes the following supplementary texts, tables and references:**

**Text S1.** Literature review of crossing compatibility and phylogenetic analysis to develop four gene pools

**Text S2.** Selected bioclimatic variables for Maxent distribution modelling

**Text S3.** R packages used

**Table S1.** Accepted *Vigna* names and taxonomic classification following GRIN taxonomy ^1^ and Iseki et al. ^2^

**Table S2.** Biotic stress tolerance of *Vigna* species

**Table S3.** Reported accessions conserved *ex situ* in 2017

**Table S4.** Targeted countries for collecting of *V.* species with less than 10 genebank accessions

**References**

**Text S1.** Literature review of crossing compatibility and phylogenetic analysis to develop four gene pools

**Domesticated *Vigna* taxa**

**Gene pool A. Subgenus *Ceratotropis*:** *Vigna aconitifolia* ^3,4^, *V. angularis* ^3,4^, *V. mungo* ^3,4^, *V. radiata*^3,4^, *V. reflexo-pilosa* var. *glabra* ^3,4^, *V. umbellata*^3,4^.

**Gene pool B. Subgenus *Plectotropis*:** *V. vexillata* ^3,4^.

**Gene pool C. Subgenus *Vigna*:** *V. unguiculata* goups ^4^

**Gene pool D. subgenus *Vigna*:** *V. subterranea* ^4^.

**Gene pool A. Subgenus *Ceratotropis***

*Vigna angularis* x *V. hirtella*: secondary gene pool. Fertile F1 hybrids were produced between male *V. angularis* and female *V. hirtella* ^5,6^.

*Vigna angularis* x *V. minima*, *V. nakashimae*, *V. riukiuensis, V. tenuicaulis*: secondary gene pool. Fertile F1 hybrids were developed in all cases ^6^.

*Vigna angularis* x *V. nepalensis*: primary gene pool. Fertile F1 hybrids were developed ^6^.

*Vigna angularis* x *V. umbellata:* secondary gene pool. Only crosses with female *V. umbellata* plants produced fertile F1 hybrids ^6,7^.

*Vigna radiata* x *V. mungo*: secondary gene pool. Hybrids between *V. radiata* and *V. mungo* could be produced when *V. radiata* is the female parent. F1 seeds often shrivelled and did not germinate. F1 plants showed low fertility^6,8^.

*Vigna radiata* x *V. radiata* var. *sublobata*: primary gene pool. Crosses produced fertile F1, although in some crosses there was reduced F1 pollen sustainability ^6,9^.

*Vigna radiata* x *V. trilobata*: tertiary gene pool. Crosses with female and male *V. radiata* plants returned low pod set and low germination rates ^10^. Successful F1 hybrids were made (person communication Ram Nair, World Vegetable Center). Moderate crossability was observed between *V. radiata* and *V. trilobata* (8.48%) ^11^.

*Vigna radiata* x *V. vexillata*: tertiary gene pool*.* Crosses with female and male *V. radiata* plants returned low pod set and low germination rates ^10^.

*Vigna radiata* x *V. grandiflora*: tertiary gene pool. Embryo rescue was necessary to obtain F1 hybrids ^6,12^.

*Vigna radiata* and *V. mungo* x *V. subramaniana*: tertiary gene pool. Embryo rescue was necessary to obtain F1 hybrids ^5,6^.

*Vigna radiata* x *V. aconitifolia*: tertiary gene pool. Moderate crossability was observed between female *V. radiata* and male *V. trilobata* with good seed production (8.48%) ^11^.

*Vigna radiata* x *V. stipulacea*: tertiary gene pool. Low podset between female V. radiata and male *V. stipulacea* ^10^*.*

*V. radiata x Vigna umbellata*: tertiary gene pool. Sterile F1 hybrids were produced by crossing female *V. radiata* and male *V. umbellata* ^6,13^.

*Vigna radiata* x *V. reflexo-pilosa* var. *glabra*: tertiary gene pool. Crosses between male *V. radiata* and female *V. reflexo-pilosa* var. *glabra* returned sterile F1 hybrids ^6,14^.

*Vigna mungo x V. mungo var. silvestris*: primary gene pool. Crosses produced fertile F1, although in some crosses there is reduced F1 pollen sustainability ^6,9^.

*Vigna umbellata* x *V. minima*, *V. nakashimae*, *V. riukiuensis, V. nepalensis, V. tenuicaulis*: secondary gene pool. Only crosses with female *V. umbellata* plants produced fertile F1 hybrids ^6^.

*Vigna umbellata* x *V. minima:* tertiary gene pool. Crosses produced sterile F1. V. minima was considered as the tertiary gene pool of the rice bean ^6,15^.

*Vigna minima* x *V. hirtella*. Primary gene pool. Natural hybridization results in prima fertile F1 ^3^.

**Gene pool B. Subgenus *Plectotropis***

*Vigna vexillata* x *Vigna vexillata* var. *davyi*: secondary gene pool. Crosses between pairs were partially fertile ^16^.

*Vigna vexillata* x *Vigna vexillata* var. *angustifolia*: secondary gene pool. Pollen fertility between 47% ^16^.

*Vigna vexillata* x *Vigna vexillata* var. *vexillata*: secondary gene pool. Pollen fertility between 59% ^16^.

*Vigna vexillata* x *Vigna unguiculata:* seperate gene pools. No viable hybrids even not after embryo rescue ^17^.

**Gene pool C. Subgenus *Vigna***

*Vigna schlechteri* and *V. keraudrenii* are part of the secondary gene pool of *V. unguiculata* ^18^*.*

*Vigna unguiculata* x *Vigna unguiculata* ssp. *stenophylla:* secondary gene pool. The F1 hybrids of crossings between these two species were partially fertile ^16^.

*Vigna unguiculata* x *Vigna unguiculata* ssp. d*ekindtiana:* tertiary gene pool. A successful cross was made with the help of embryo rescue ^16^.

**Gene pool D. subgenus *Vigna***

*Vigna oblongifolia* x *V. luteola:* tertiary gene pool. A successful cross was made with the help of embryo rescue ^16^.

**Additional species were added to the gene pool model based on taxonomic and phylogenetic analysis:**

**Gene pool A. *Ceratotropis*:** *Vigna dalzelliana* ^2^; *V. hainiana* ^19^; *Vigna indica* ^2^; *V. khandalensis* ^2^; *V. nepalensis* ^19,20^; *V. sahyadriana* ^2^; *V. stipulacea* ^19^; and *V. trinervia* ^2,19^*.*

**Gene pool B. *Plectotropics*:** *Vigna angivensis* ^21^; *V. lobatifolia* ^22^; and *V. vexilliata* spp. ^2,19,21^*.*

**Gene pool C. *Vigna*:** *Vigna friesiorum* ^23^; *V. frutescens* ssp ^21,23^; *V. keraudrenii* ^4^;  *V. reticulata* ^21^; and *V. schlechteri* ^4,18^*.*

**Gene pool D. *Vigna*:** *Vigna ambacensis* ^21^; *V. fischeri* ^23^; *V. gracilis* ^21^; *V. heterophylla* ^21^; *V. hosei* ^4,21^; *V. laurentii* ^21^; *Vigna luteola* ^2,21,24^; *Vigna marina* ^2,21,24^; *V. membranacea* ^21,23^; *V. multinervis* ^21,25^; *V. parkeri* ssp*.* ^21^; *V. racemosa* ^21,25^; and *V. subterranea* ^2,24^*.*

**Text S2.** Selected bioclimatic variables for Maxent distribution modelling

- Mean Diurnal Range (Mean of monthly (max temp - min temp));
- Mean Temperature of Warmest Quarter;
- Mean Temperature of Coldest Quarter;
- Annual Precipitation;
- Precipitation of Driest Month;
- Precipitation Seasonality (Coefficient of Variation);
- Precipitation of Warmest Quarter; and
- Precipitation of Coldest Quarter.

**Text S3.** R packages used

The following R packages were used in the collection of presence records, Maxent distribution modelling, and the ecogeographic analysis: rgbif´ ^26^, `raster´^27^, `dismo´ ^28^, `sp´ ^29^, `rgeos´ ^30^, `rgdal´ ^31^, `geosphere´^32^ , and `maptools´ ^33^, `ggplot´ ^34^, `doBy´ ^35^, and `agricolae´ ^36^.

**Table S1.** Accepted *Vigna* names and taxonomic classification following GRIN taxonomy ^1^ and Iseki et al. ^2^

| Subgenus | Section | Species | Complete scientific name | Taxonomy |
| --- | --- | --- | --- | --- |
| *Ceratotropis* | *Angulares* | *V. angularis* | *V. angularis* (Willd.) Ohwi & H. Ohashi | ^1^ |
| *Ceratotropis* | *Angulares* | *V. angularis* var. *nipponensis* | *V. angularis* var. *nipponensis* (Ohwi) Ohwi & H. Ohashi | ^1^ |
| *Ceratotropis* | *Angulares* | *V. dalzelliana* | *V. dalzelliana* (Kuntze) Verdc. | ^1^ |
| *Ceratotropis* | *Angulares* | *V. exilis* | *V. exilis* Tateishi & Maxted | ^1^ |
| *Ceratotropis* | *Angulares* | *V. hirtella* | *V. hirtella* Ridl. | ^1^ |
| *Ceratotropis* | *Angulares* | *V. minima* | *V. minima* (Roxb.) Ohwi & H. Ohashi | ^1^ |
| *Ceratotropis* | *Angulares* | *V. nakashimae* | *V. nakashimae* (Ohwi) Ohwi & H. Ohashi | ^1^ |
| *Ceratotropis* | *Angulares* | *V. nepalensis* | *V. nepalensis* Tateishi & Maxted | ^1^ |
| *Ceratotropis* | *Angulares* | *V. reflexopilosa* | *V. reflexopilosa* Hayata | ^1^ |
| *Ceratotropis* | *Angulares* | *V. reflexopilosa*subsp.*glabra* | *V. reflexopilosa* subsp. glabra (Roxb.) N. Tomooka & Maxted | ^1^ |
| *Ceratotropis* | *Angulares* | *V. riukiuensis* | *V. riukiuensis* (Ohwi) Ohwi & H. Ohashi | ^1^ |
| *Ceratotropis* | *Angulares* | *V. tenuicaulis* | *V. tenuicaulis* N. Tomooka & Maxted | ^1^ |
| *Ceratotropis* | *Angulares* | *V. trinervia* | *V. trinervia* (B. Heyne ex Wight & Arn.) Tateishi & Maxted | ^1^ |
| *Ceratotropis* | *Angulares* | *V. umbellata* | *V. umbellata* (Thunb.) Ohwi & H. Ohashi | ^1^ |
| *Ceratotropis* | *Ceratotropis* | *V. grandiflora* | *V. grandiflora* (Prain) Tateishi & Maxted | ^1^ |
| *Ceratotropis* | *Ceratotropis* | *V. hainiana* |  | ^2^ |
| *Ceratotropis* | *Ceratotropis* | *V. mungo* | *V. mungo* (L.) Hepper | ^1^ |
| *Ceratotropis* | *Ceratotropis* | *V. mungo* var. *silvestris* | *V. mungo* var. *silvestris* Lukoki et al. | ^1^ |
| *Ceratotropis* | *Ceratotropis* | *V. radiata* | *V. radiata* (L.) R. Wilczek | ^1^ |
| *Ceratotropis* | *Ceratotropis* | *V. radiata*var.*sublobata* | *V. radiata* var. *sublobata* (Roxb.) Verdc. | ^1^ |
| *Ceratotropis* | *Ceratotropis* | *V. sahyadriana* |  | ^2^ |
| *Ceratotropis* | *Aconitifoliae* | *V. aconitifolia* | *V. aconitifolia* (Jacq.) Maréchal | ^1^ |
| *Ceratotropis* | *Aconitifoliae* | *V. aridicola* | *V. aridicola* N. Tomooka & Maxted | ^1^ |
| *Ceratotropis* | *Aconitifoliae* | *V. indica* |  | ^2^ |
| *Ceratotropis* | *Aconitifoliae* | *V. khandalensis* | *V. khandalensis* (Santapau) Sundararagh. & Wadhwa | ^1^ |
| *Ceratotropis* | *Aconitifoliae* | *V. stipulacea* | *V. stipulacea* (Lam.) Kuntze | ^1^ |
| *Ceratotropis* | *Aconitifoliae* | *V. subramaniana* | *V. subramaniana* (Babu ex Raizada) Raizada | ^1^ |
| *Ceratotropis* | *Aconitifoliae* | *V. trilobata* | *V. trilobata* (L.) Verdc. | ^1^ |
| *Plectrotropis* | *Plectotropis* | *V. kirkii* | *V. kirkii* (Baker) J. B. Gillett | ^1^ |
| *Plectrotropis* | *Plectotropis* | *V. vexillata* | *V. vexillata* (L.) A. Rich. | ^1^ |
| *Plectrotropis* | *Plectotropis* | *V. vexillata*var.*angustifolia* | *V. vexillata* var. *angustifolia* (Schumach.) Baker | ^1^ |
| *Plectrotropis* | *Plectotropis* | *V. vexillata*var.*davyi* | *V. vexillata* var. *davyi* (Bolus) B. J. Pienaar | ^1^ |
| *Plectrotropis* | *Plectotropis* | *V. vexillata*var.*macrosperma* | *V. vexillata* var. *macrosperma* Maréchal et al. | ^1^ |
| *Plectrotropis* | *Plectotropis* | *V. vexillata*var.*ovata* | *V. vexillata* var. *ovata* (E. Mey.) B. J. Pienaar, nom. inval. | ^1^ |
| *Plectrotropis* | *Plectotropis* | *V. vexillata*var.*vexillata* | *V. vexillata* var. *vexillat*a | ^1^ |
| *Plectrotropis* | *Plectotropis* | *V. vexillata*var.*youngiana* | *V. vexillata* var. *youngiana* F. M. Bailey | ^1^ |
| *Plectrotropis* | *Pseudoliebrechtsia* | *V. lobatifolia* | *V. lobatifolia* Baker | ^1^ |
| *Vigna* | *Catiang* | *V. keraudrenii* | *V. keraudrenii* Du Puy & Labat | ^1^ |
| *Vigna* | *Catiang* | *V. monantha* | *V. monantha* Thulin | ^1^ |
| *Vigna* | *Catiang* | *V. schlechteri* | *V. schlechteri* Harms | ^1^ |
| *Vigna* | *Catiang* | *V. unguiculata* | *V. unguiculata* (L.) Walp. | ^1^ |
| *Vigna* | *Catiang* | *V. unguiculata* group*biflora* | *V. unguiculata* (L.) Walp. group *biflora* | ^1^ |
| *Vigna* | *Catiang* | *V. unguiculata* subsp.*baoulensis* | *V. unguiculata* (L.) Walp. subsp. *baoulensis* (A. Chev.) Pasquet | ^1^ |
| *Vigna* | *Catiang* | *V. unguiculata* subsp.*protracta* | *V. unguiculata* (L.) Walp. subsp. *protracta* (E. Mey.) B. J. Pienaar | ^1^ |
| *Vigna* | *Catiang* | *V. unguiculata*group*melanophthalmus* | *V. unguiculata* (L.) Walp. group *melanophthalmu*s | ^1^ |
| *Vigna* | *Catiang* | *V. unguiculata*group*unguiculata* | *V. unguiculata* (L.) Walp. group *unguiculata* | ^1^ |
| *Vigna* | *Catiang* | *V. unguiculata*subsp.*aduensis* | *V. unguiculata* subsp. *aduensis*Pasquet | ^1^ |
| *Vigna* | *Catiang* | *V. unguiculata*subsp.*burundiensis* | *V. unguiculata* (L.) Walp. subsp. *burundiensis* Pasquet | ^1^ |
| *Vigna* | *Catiang* | *V. unguiculata subsp. letouzeyi* | *V. unguiculata* (L.) Walp. subsp. *letouzeyi* Pasquet | ^1^ |
| *Vigna* | *Catiang* | *V. unguiculata subsp. pawekiae* | *V. unguiculata* (L.) Walp. subsp. *pawekiae* Pasquet | ^1^ |
| *Vigna* | *Catiang* | *V. unguiculata*subsp.*tenuis* | *V. unguiculata* subsp. *tenuis* (E. Mey.) Maréchal et al. | ^1^ |
| *Vigna* | *Catiang* | *V. unguiculata*subsp.*unguiculata* | *V. unguiculata* subsp. *unguiculata* | ^1^ |
| *Vigna* | *Catiang* | *V. unguiculata*subsp. *stenophylla* | *V. unguiculata* subsp. *stenophylla* (Harv.) Maréchal et al. | ^1^ |
| *Vigna* | *Catiang* | *V. unguiculata*var*. spontanea* | *V. unguiculata* var. *spontanea* (Schweinf.) Pasquet | ^1^ |
| *Vigna* | *Catiang* | *V. unguiculata*group*sesquipedalis* | *V. unguiculata* group *sesquipedalis* | ^1^ |
| *Vigna* | *Catiang* | *V. unguiculata*subsp.*alba* | *V. unguiculata* subsp*. alba* (G. Don) Pasquet | ^1^ |
| *Vigna* | *Catiang* | *V. unguiculata*subsp.*dekindtiana* | *V. unguiculata* subsp. *dekindtiana* (Harms) Verdc. | ^1^ |
| *Vigna* | *Catiang* | *V. unguiculata*subsp*. pubescens* | *V. unguiculata* subsp. *pubescens* (R. Wilczek) Pasquet | ^1^ |
| *Vigna* | *Macrodontae* | *V. friesiorum* | *V. friesiorum* Harms | ^1^ |
| *Vigna* | *Macrodontae* | *V. frutescens* | *V. frutescens* A. Rich. | ^1^ |
| *Vigna* | *Macrodontae* | *V. frutescens*subsp.*incana* | *V. frutescens* subsp.*incana* (Taub.) Verdc. | ^1^ |
| *Vigna* | *Macrodontae* | *V. frutescens*var.*buchneri* | *V. frutescens* var. *buchneri*(Harms) Verdc. | ^1^ |
| *Vigna* | *Macrodontae* | *V. membranacea* | *V. membranacea* A. Rich. | ^1^ |
| *Vigna* | *Macrodontae* | *V. somaliensis* | *V. somaliensis* Baker f. | ^1^ |
| *Vigna* | *Reticulatae* | *V. reticulate* | *V. reticulata* Hook. f. | ^1^ |
| *Vigna* | *Reticulatae* | *V. wittei* | *V. wittei* Baker f. | ^1^ |
| *Vigna* | *Vigna* | *V. ambacensis* | *V. ambacensis* Welw. ex Baker | ^1^ |
| *Vigna* | *Vigna* | *V. angivensis* | *V. angivensis* Baker | ^1^ |
| *Vigna* | *Vigna* | *V. bequaertii* | *V. bequaertii* R. Wilczek | ^1^ |
| *Vigna* | *Vigna* | *V. comosa* | *V. comosa* Baker | ^1^ |
| *Vigna* | *Vigna* | *V. comosa*var.*lebrunii* | *V. comosa* var. *lebruni*i | ^1^ |
| *Vigna* | *Vigna* | *V. filicaulis* | *V. filicaulis* Hepper | ^1^ |
| *Vigna* | *Vigna* | *V. filicaulis* var*. pseudovenulosa* | *V. filicaulis* var. *pseudovenulosa* Maréchal et al. | ^1^ |
| *Vigna* | *Vigna* | *V. fischeri* | *V. fischeri* Harms | ^1^ |
| *Vigna* | *Vigna* | *V. gazensis* | *V. gazensis* Baker f. | ^1^ |
| *Vigna* | *Vigna* | *V. gracilis* | *V. gracilis* (Guill. & Perr.) Hook. f. | ^1^ |
| *Vigna* | *Vigna* | *V. heterophylla* | *V. heterophylla* A. Rich. | ^1^ |
| *Vigna* | *Vigna* | *V. hosei* | *V. hosei* (Craib) Backer | ^1^ |
| *Vigna* | *Vigna* | *V. laurentii* | *V. laurentii* De Wild. | ^1^ |
| *Vigna* | *Vigna* | *V. luteola* | *V. luteola* (Jacq.) Benth. | ^1^ |
| *Vigna* | *Vigna* | *V. marina* | *V. marina* (Burm.) Merr. | ^1^ |
| *Vigna* | *Vigna* | *V. multinervis* | *V. multinervis* Hutch. & Dalziel | ^1^ |
| *Vigna* | *Vigna* | *V. oblongifolia* | *V. oblongifolia* A. Rich. | ^1^ |
| *Vigna* | *Vigna* | *V. o-wahuensis* | *V. o-wahuensis* Vogel | ^1^ |
| *Vigna* | *Vigna* | *V. parkeri* | *V. parkeri* Baker | ^1^ |
| *Vigna* | *Vigna* | *V. parkeri*subsp.*acutifolia* | *V. parkeri* subsp*. acutifolia* Verdc. | ^1^ |
| *Vigna* | *Vigna* | *V. parkeri*subsp.*maranguensis* | *V. parkeri* subsp. *maranguensi*s (Taub.) Verdc. | ^1^ |
| *Vigna* | *Vigna* | *V. racemosa* | *V. racemosa* (G. Don) Hutch. & Dalziel | ^1^ |
| *Vigna* | *Vigna* | *V. subterranea* | *V. subterranea* (L.) Verdc. | ^1^ |
| *Vigna* | *Vigna* | *V. subterranea*var.*spontanea* | *V. subterranea* var. *spontanea* (Harms) Pasquet | ^1^ |
| *Vigna* | *Vigna* | *V. oblongifolia*var.*parviflora* | *V. oblongifolia* A. Rich. var. *parviflora* (Welw. ex Baker) Verdc. | ^1^ |

**Table S2.** Biotic stress resistance of *Vigna* species

| Gene  pool | Subgenus | Section | Species | Insect and disease resistance found | References |
| --- | --- | --- | --- | --- | --- |
| A | *Ceratotropis* | *Aconitifoliae* | *V. aconitifolia* | YMD | ^37^ |
| A | *Ceratotropis* | *Aconitifoliae* | *V. subramaniana* | Bruchids (*C. chinensis*) | ^38^ |
| A | *Ceratotropis* | *Aconitifoliae* | *V. trilobata* | YMD | ^39^ |
| A | *Ceratotropis* | *Angulares* | *V. dalzelliana* | YMD | ^39^ |
| A | *Ceratotropis* | *Angulares* | *V. hirtella* | Bruchids (*Callosobruchus chinensis* and *C. macualtus*) | ^38^ |
| A | *Ceratotropis* | *Angulares* | *V. minima* | Bruchids (*C. chinensis* and *C. macualtus*) | ^40^ |
| A | *Ceratotropis* | *Angulares* | *V. nepalensis* | Bruchids (*C. chinensis* and *C. macualtus*) | ^38,39^ |
| A | *Ceratotropis* | *Angulares* | *V. reflexopilosa* | Bean fly (*Ophiomyia* phaseoli; *O. centrosematis*; *Melanagromyza sojae*); Bruchids (*Callosobruchus* spp.) | ^39,41^ |
| A | *Ceratotropis* | *Angulares* | *V. riukiuensis* | Bruchids (*Callosobruchus* spp.) | ^41^ |
| A | *Ceratotropis* | *Angulares* | *V. tenuicaulis* | Bruchids (*Callosobruchus* spp.) | ^40^ |
| A | *Ceratotropis* | *Angulares* | *V. trinervia* | YMD | ^39^ |
| A | *Ceratotropis* | *Angulares* | *V. umbellata* | Bruchids (*Callosobruchus* spp.); YMD | ^39,40^ |
| A | *Ceratotropis* | *Angulares* | *V. reflexopilosa*ssp.*glabra* | Bruchids (*Callosobruchus* spp.); bean fly; YMD; CMV; Powdery mildew | ^12,39^ |
| A | *Ceratotropis* | *Angulares* | *V. trinervia* | Bruchids (*C. chinensis*) | ^38^ |
| A | *Ceratotropis* | *Ceratotropis* | *V. radiata* | Anthracnose (*Colletotrichum lindemuthianum* or *C. truncatum* or *C. gloeosporioides*); Bean blossom thrips (*Megalurothrips distalis)*; Cotton bollworm (*Helicoverpa armigera*); Cowpea aphid (*Aphis craccivora*); Cercospora leaf spot (*Cercospora cruenta* or *C. canescens* or *C. kikuchii* or *C. caracallae*); Dry root rot (*Rhizoctonia bataticola*); Green Jassid (*Empoasca* spp.); Legume pod borer (*Maruca vitrata*); Macrophomina blight (*M. phaseolina*); Powdery mildew (*Erysiphe polygoni* or *Podosphaera fusca*); Stem borer (*Ophiomyia* spp.); Whitefly (*Bemisia tabaci*); YMD | ^39,42,43^ |
| A | *Ceratotropis* | *Ceratotropis* | *V. mungo* | Bean blossom thrips (*M. distalis*); Bruchids (*C. chinensis*); Cotton bollworm (*H. armigera*); Cowpea aphid (*A. craccivora*); Whitefly (*B. tabaci*); YMD | ^43,44^ |
| A | *Ceratotropis* | *Ceratotropis* | *V. mungo* var. *silvestris* | Bruchids (*Callosobruchus* spp.) | ^39^ |
| A | *Ceratotropis* | *Ceratotropis* | *V. radiata* var. *sublobata* | Bruchids (*Callosobruchus* spp.); YMD | ^39^ |
| B | *Plectrotropis* | *Plectotropis* | *V. vexillata* | Bruchids (*C. maculatus*); CPMoV | ^39^ |
| C | *Vigna* | *Catiang* | *V. unguiculata* | Anthracnose (*Colletotrichum destructivum*); Bacterial blight (*Xanthomonas vignicola* Burkh.); Bean Bug (*Clavigralla tomentosicollis*); Bean fly (*O. phaseoli*); BICMV; Bruchids (*C. maculatus*); CAMV; Cowpea aphid (*A. craccivora*); Cowpea flower thrips (*M. sjostedti*); Leafhoppers (*Empoasca* spp.); Legume pod borer (*M. vitrata);* Root‑knot nematodes *(Meloidogyne* spp.); Thrips (*Frankliniella* spp.); YMD | ^44–47^ |
| C | *Vigna* | *Catiang* | *V. unguiculata*ssp.*mensensis* | Cowpea moth (*Cydia ptychor*) | ^39^ |
| C | *Vigna* | *Catiang* | *V. unguiculata* ssp. *dekindtiana* | Coreid bug (*Clavigralla tomentosicollis*) | ^39^ |
| C | *Vigna* | *Reticulatae* | *V. reticulata* | Bruchids (*C. maculatus*) | ^48^ |
| D | *Vigna* | *Vigna* | *V. luteola* | Bruchids (*C. maculatus*) | ^48^ |
| D | *Vigna* | *Vigna* | *V. oblongifolia* | Bruchds (*C. maculatus*) | ^48^ |
| CAMV: Cowpea aphid-borne mosaic virus; BICMV: Blackeye cowpea mosaic virus; CPMoV: Cowpea mottle carmovirus; YMD: Yellow mosaic disease viruses. | | | | | |
|  | | | | | |

**Table S3.** Reported accessions conserved *ex situ* in 2017

| Gene  pool | Subgenus | Section | | Species | Meise, Belgium | | IITA | | Australian Grains Genebank | | CIAT | | NARO, Japan | | NBPGR, India | | WorldVeg | | Subtotal | | Total | |  |
| --- | --- | --- | --- | --- | --- | --- | --- | --- | --- | --- | --- | --- | --- | --- | --- | --- | --- | --- | --- | --- | --- | --- | --- |
| A | *Ceratotropis* | *Angulares* | | *V. angularis* | 9 | |  | | 349 | | 3 | | 1492 | | 175 | | 2350 | | 4378 | | 5082 | |  |
| A | *Ceratotropis* | *Angulares* | | *V. dalzelliana* |  | |  | | 4 | | 4 | |  | | 21 | |  | | 29 | | 36 | |  |
| A | *Ceratotropis* | *Angulares* | | *V. exilis* | 1 | |  | |  | |  | |  | |  | |  | | 1 | | 1 | |  |
| A | *Ceratotropis* | *Angulares* | | *V. hirtella* | 3 | |  | |  | |  | | 4 | |  | |  | | 7 | | 7 | |  |
| A | *Ceratotropis* | *Angulares* | | *V. minima* | 2 | | 1 | |  | | 2 | | 5 | | 1 | |  | | 11 | | 12 | |  |
| A | *Ceratotropis* | *Angulares* | | *V. nakashimae* | 2 | |  | |  | |  | | 21 | |  | |  | | 23 | | 24 | |  |
| A | *Ceratotropis* | *Angulares* | | *V. nepalensis* | 3 | |  | |  | |  | | 4 | | 3 | |  | | 10 | | 10 | |  |
| A | *Ceratotropis* | *Angulares* | | *V. reflexopilosa* | 3 | | 2 | | 1 | | 2 | | 37 | |  | | 3 | | 48 | | 50 | |  |
| A | *Ceratotropis* | *Angulares* | | *V. riukiuensis* | 1 | |  | |  | |  | | 63 | |  | |  | | 64 | | 64 | |  |
| A | *Ceratotropis* | *Angulares* | | *V. tenuicaulis* | 1 | |  | |  | |  | | 2 | |  | |  | | 3 | | 3 | |  |
| A | *Ceratotropis* | *Angulares* | | *V. trinervia* | 2 | |  | |  | |  | | 6 | | 8 | |  | | 16 | | 16 | |  |
| A | *Ceratotropis* | *Angulares* | | *V. umbellata* | 13 | | 1 | | 59 | | 39 | | 214 | | 2050 | | 320 | | 2696 | | 3003 | |  |
| A | *Ceratotropis* | *Ceratotropis* | | *V. grandiflora* | 1 | |  | |  | |  | | 1 | |  | |  | | 2 | | 2 | |  |
| A | *Ceratotropis* | *Ceratotropis* | | *V. hainiana* |  | |  | |  | |  | |  | | 2 | |  | | 2 | | 2 | |  |
| A | *Ceratotropis* | *Ceratotropis* | | *V. mungo* | 12 | | 11 | | 104 | | 96 | | 145 | | 1751 | | 849 | | 2968 | | 5987 | |  |
| A | *Ceratotropis* | *Ceratotropis* | | *V. radiata* | 34 | | 124 | | 1385 | | 69 | | 922 | | 4024 | | 6752 | | 13310 | | 21161 | |  |
| A | *Ceratotropis* | *Ceratotropis* | | *V. sahyadriana* |  | |  | |  | |  | |  | |  | |  | | 0 | | 0 | |  |
| A | *Ceratotropis* | *Aconitifoliae* | | *V. aconitifolia* | 7 | |  | | 35 | | 8 | | 6 | | 1486 | | 26 | | 1568 | | 1849 | |  |
| A | *Ceratotropis* | *Aconitifoliae* | | *V. aridicola* | 1 | |  | |  | |  | |  | |  | |  | | 1 | | 1 | |  |
| A | *Ceratotropis* | *Aconitifoliae* | | *V. indica* |  | |  | |  | |  | |  | |  | |  | | 0 | | 0 | |  |
| A | *Ceratotropis* | *Aconitifoliae* | | *V. khandalensis* |  | |  | |  | |  | |  | | 1 | |  | | 1 | | 1 | |  |
| A | *Ceratotropis* | *Aconitifoliae* | | *V. stipulacea* | 4 | |  | |  | |  | | 1 | |  | |  | | 5 | | 5 | |  |
| A | *Ceratotropis* | *Aconitifoliae* | | *V. subramaniana* | 1 | |  | | 2 | |  | | 1 | |  | |  | | 4 | | 6 | |  |
| A | *Ceratotropis* | *Aconitifoliae* | | *V. trilobata* | 4 | | 7 | | 53 | | 4 | |  | | 130 | | 2 | | 200 | | 336 | |  |
| B | *Plectrotropis* | *Plectotropis* | | *V. kirkii* | 1 | | 6 | | 1 | |  | |  | |  | |  | | 8 | | 9 | |  |
| B | *Plectrotropis* | *Plectotropis* | | *V. vexillata* | 135 | | 195 | | 187 | | 201 | | 6 | | 110 | | 2 | | 836 | | 1068 | |  |
| B | *Plectrotropis* | *Pseudoliebrechtsia* | | *V. lobatifolia* |  | | 3 | | 1 | |  | |  | |  | |  | | 4 | | 5 | |  |
| C | *Vigna* | *Catiang* | | *V. keraudrenii* |  | |  | |  | |  | |  | |  | |  | | 0 | | 0 | |  |
| C | *Vigna* | *Catiang* | | *V. monantha* |  | |  | |  | |  | |  | |  | |  | | 0 | | 0 | |  |
| C | *Vigna* | *Catiang* | | *V. slechteri* |  | | 9 | |  | |  | |  | |  | |  | | 9 | | 9 | |  |
| C | *Vigna* | *Catiang* | | *V. unguiculata* | 332 | | 16127 | | 935 | | 94 | | 1371 | | 3649 | | 1610 | | 24118 | | 44694 | |  |
| C | *Vigna* | *Macrodontae* | | *V. friesiorum* | 1 | | 6 | | 1 | | 1 | |  | |  | |  | | 9 | | 11 | |  |
| C | *Vigna* | *Macrodontae* | | *V. frutescens* | 14 | | 7 | | 10 | | 3 | |  | |  | |  | | 34 | | 46 | |  |
| C | *Vigna* | *Macrodontae* | | *V. membranacea* | 32 | | 13 | | 3 | | 1 | |  | |  | |  | | 49 | | 96 | |  |
| C | *Vigna* | *Macrodontae* | | *V. somaliensis* |  | |  | |  | |  | |  | |  | |  | | 0 | | 0 | |  |
| C | *Vigna* | *Reticulatae* | | *V. reticulata* | 28 | | 116 | | 3 | |  | |  | |  | |  | | 147 | | 202 | |  |
| C | *Vigna* | *Reticulatae* | | *V. wittei* |  | | 29 | |  | | 1 | |  | |  | |  | | 30 | | 30 | |  |
| D | *Vigna* | *Vigna* | | *V. ambacensis* | 36 | | 151 | | 4 | | 1 | |  | |  | |  | | 192 | | 195 | |  |
| D | *Vigna* | *Vigna* | | *V. angivensis* | 3 | | 1 | | 1 | |  | |  | |  | |  | | 5 | | 6 | |  |
| D | *Vigna* | *Vigna* | | *V. bequaertii* |  | |  | |  | |  | |  | |  | |  | | 0 | | 0 | |  |
| D | *Vigna* | *Vigna* | | *V. comosa* | 1 | | 11 | |  | |  | |  | |  | |  | | 12 | | 13 | |  |
| D | *Vigna* | *Vigna* | | *V. filicaulis* | 5 | | 4 | | 2 | |  | |  | |  | |  | | 11 | | 14 | |  |
| D | *Vigna* | *Vigna* | | *V. fischeri* |  | | 1 | |  | |  | |  | |  | |  | | 1 | | 1 | |  |
| D | *Vigna* | *Vigna* | | *V. gazensis* |  | |  | |  | |  | |  | |  | |  | | 0 | | 0 | |  |
| D | *Vigna* | *Vigna* | | *V. gracilis* | 14 | | 24 | |  | |  | |  | |  | |  | | 38 | | 49 | |  |
| D | *Vigna* | *Vigna* | | *V. heterophylla* | 2 | | 1 | | 4 | |  | |  | |  | |  | | 7 | | 32 | |  |
| D | *Vigna* | *Vigna* | | *V. hosei* | 5 | | 44 | | 4 | | 7 | |  | |  | |  | | 60 | | 65 | |  |
| D | *Vigna* | *Vigna* | | *V. laurentii* | 2 | | 1 | | 1 | |  | |  | |  | |  | | 4 | | 6 | |  |
| D | *Vigna* | *Vigna* | | *V. luteola* | 24 | | 66 | | 39 | | 69 | |  | |  | | 4 | | 202 | | 257 | |  |
| D | *Vigna* | *Vigna* | | *V. marina* | 6 | | 8 | | 39 | |  | | 1 | | 2 | | 43 | | 99 | | 129 | |  |
| D | *Vigna* | *Vigna* | | *V. multinervis* | 5 | | 17 | |  | |  | |  | |  | |  | | 22 | | 23 | |  |
| D | *Vigna* | *Vigna* | | *V. oblongifolia* | 31 | | 55 | | 42 | | 31 | |  | |  | |  | | 159 | | 256 | |  |
| D | *Vigna* | *Vigna* | | *V. o-wahuensis* | 1 | |  | |  | |  | |  | |  | |  | | 1 | | 1 | |  |
| D | *Vigna* | *Vigna* | | *V. parkeri* | 6 | | 2 | | 20 | | 1 | |  | |  | | 1 | | 30 | | 80 | |  |
| D | *Vigna* | *Vigna* | | *V. racemosa* | 43 | | 131 | | 6 | | 1 | |  | |  | |  | | 181 | | 208 | |  |
| D | *Vigna* | *Vigna* | | *V. subterranea* |  | | 2088 | | 47 | |  | |  | |  | |  | | 2135 | | 4125 | |  |
|  |  | | Number of accessions | | | 831 | | 19262 | | 3342 | | 638 | | 4302 | | 13413 | | 11962 | | 53750 | | 89288 | |
|  |  | | Number of taxa | | | 41 | | 32 | | 29 | | 21 | | 19 | | 15 | | 12 | | 55 | | 55 | |

**Table S4.** Targeted countries for collecting of *Vigna* species with less than 10 genebank accessions

| Subgenus | Section | Taxa | Reported occurrence | Modelled occurrence |
| --- | --- | --- | --- | --- |
| *Ceratotropis* | *Angulares* | *V. exilis* | **Thailand ^i^** | **Thailand**, Myanmar |
| *Ceratotropis* | *Angulares* | *V. hirtella* | **Thailand**, India, Myanmar, Malaysia, Vietnam, China, Lao People's Democratic Republic | **Myanmar**, Thailand, India, Cambodia, China, Malaysia, Indonesia, Bhutan, Nepal, Bangladesh |
| *Ceratotropis* | *Angulares* | *V. tenuicaulis* | **Thailand** | **Thailand** |
| *Ceratotropis* | *Ceratotropis* | *V. grandiflora* | **Thailand**, Cambodia | **Thailand**, Cambodia |
| *Ceratotropis* | *Ceratotropis* | *V. hainiana* | **India** | **India**, Nepal |
| *Ceratotropis* | *Ceratotropis* | *V. sahyadriana* | na | na |
| *Ceratotropis* | *Aconitifoliae* | *V. aridicola* | **Sri Lanka** | **Sri Lanka** |
| *Ceratotropis* | *Aconitifoliae* | *V. indica* | na | na |
| *Ceratotropis* | *Aconitifoliae* | *V. khandalensis* | **India** | **India** |
| *Ceratotropis* | *Aconitifoliae* | *V. stipulacea* | **India**, Indonesia, Sri Lanka, Spain, Vietnam | **India**, Thailand, Myanmar, Indonesia, Cambodia, Australia, Philippines, Sri Lanka, Ethiopia, Bangladesh, South Sudan, Malaysia, Sudan, China, Maldives |
| *Ceratotropis* | *Aconitifoliae* | *V. subramaniana* | **India** | **India**, Pakistan, Nepal, China |
| *Plectrotropis* | *Plectotropis* | *V. kirkii* | **Tanzania**, DRC Congo, Uganda, Mozambique, Malawi, Burundi, Cameroon, Guinea-Bissau, Guinea, Kenya, Senegal, South Sudan, Zambia | **DRC Congo**, Central African Republic, Mozambique, Cameroon, Congo, Nigeria, Uganda, Ghana, South Sudan, Angola, Gabon, Malawi, Kenya, Zambia, Guinea, Ethiopia, Senegal, Guinea-Bissau, Mali, Togo, Benin, Liberia, Burundi, Burkina Faso, Rwanda, Madagascar, Chad, Equatorial Guinea, Zimbabwe, Sierra Leone, Gambia, Comoros |
| *Plectrotropis* | *Pseudoliebrechtsia* | *V. lobatifolia* | **Namibia** | na |
| *Vigna* | *Catiang* | *V. keraudrenii* | **Madagascar** | **Madagascar** |
| *Vigna* | *Catiang* | *V. monantha* | **Somalia** | **Somalia** |
| *Vigna* | *Catiang* | *V. schlechteri* | **South Africa**, Zimbabwe, Swaziland, Mozambique | South Africa, Zimbabwe, Swaziland, Mozambique, Lesotho |
| *Vigna* | *Macrodontae* | *V. somaliensis* | na | na |
| *Vigna* | *Vigna* | *V. angivensis* | **Madagascar**, Burundi, Russian Federation | **Madagascar**, Nepal, India, Mozambique, Democratic Republic of the Congo, Reunion (France), Comoros, Mauritius, Zimbabwe, Kenya, China, Uganda, Malawi, Ethiopia, Rwanda |
| *Vigna* | *Vigna* | *V. bequaertii* | **DRC Congo**, Burundi, Rwanda | **DRC Congo**, Central African Republic, Rwanda, Uganda, Burundi, South Sudan, Angola |
| *Vigna* | *Vigna* | *V. fischeri* | **Burundi**, Malawi | na |
| *Vigna* | *Vigna* | *V. gazensis* | **Mozambique**, Malawi, Madagascar, Zimbabwe | **Madagascar**, Mozambique, Zimbabwe, Malawi, Zambia |
| *Vigna* | *Vigna* | *V. laurentii* | **Benin**, DRC Congo, Cameroon, Burundi, Togo, Central African Republic, Gabon, Guinea-Bissau, South Sudan | **DRC Congo**, Nigeria, Central African Republic, Cameroon, Angola, South Sudan, Zambia, Ghana, Gabon, Congo, Uganda, Chad, Benin, Togo, Guinea, Burundi, Guinea-Bissau, Rwanda, Burkina Faso, Senegal, Mali, Equatorial Guinea, Sudan, Kenya, Sao Tome and Principe, Malawi, Sierra Leone |
| *Vigna* | *Vigna* | *V. o-wahuensis* | **United States** | **United States**, Canada, Mexico |
| ^i^ Countries are presented in order of occurrence**. Countries in bold** reported most records of the corresponding species and most coverage of its modelled distribution | | | | |

**References**

1. USDA, ARS & NPGS. GRIN-Taxonomy. (2018). Available at: https://npgsweb.ars-grin.gov/gringlobal/taxon/taxonomysearch.aspx. (Accessed: 10th December 2018)

2. Iseki, K., Takahashi, Y., Muto, C., Naito, K. & Tomooka, N. Diversity and evolution of salt tolerance in the genus *Vigna*. *PLoS One* **11,** e0164711 (2016).

3. Tomooka, N., Isemura, T., Naito, K., Kaga, A. & Vaughan, D. *Vigna* species. in *Broadening the Genetic Base of Grain Legumes* 175–208 (Springer India, 2014). doi:10.1007/978-81-322-2023-7_9

4. Maxted, N. *et al.* *An ecogeographic study: African Vigna.* (IPGRI, 2004).

5. Tomooka, N., Egawa, Y. & Kaga, A. Biosystematics and genetic resources of the genus *Vigna* subgenus *Ceratotropis*. in *Proceedings of the 7th MAFF International Workshop on Genetic Resources. Wild Legumes* 37–62 (NIAR, 2000).

6. Tomooka, N., Vaughan, D. A., Moss, H. & Maxted, N. *The Asian Vigna: Genus Vigna subgenus Ceratotropis genetic resources*. (Springer Netherlands, 2002).

7. Chen, N. C., Baker, L. R. & Honma, S. Interspecific crossability among four species of Vigna food legumes. *Euphytica* **32,** 925–937 (1983).

8. Egawa, Y. Phylogenetic differentiation between three Asian *Vigna* species, *V. radiata*, *V. mungo* and *V. umbellata*. *Bull. Natl. Inst. Agrobiol. Resour.* **4,** 189–200 (1988).

9. Miyazaki, S. Classification and phylogenetic relationships of the *Vigna radiata*-*mungo*-*sublobata* complex. *Bull. Natl. Inst. Agric. Sci. Ser. D* **33,** 603–610 (1982).

10. Pandiyan, M. *et al.* Interspecific hybridization of *Vigna radiata* x 13 wild *Vigna* species for developing MYMV donar. *Electron. J. Plant Breed.* **4,** 600–610 (2010).

11. Bharathi, A., Vijay Selvaraj, K. S. Veerabadhiran, P. & Subba Lakshmi, B. Crossability barriers in mungbean (*Vigna radiata* L. Wilczek): with its wild relatives. *Indian J. Crop Sci.* **1,** 120–124 (2006).

12. Egawa, Y. & Tomooka, N. Phylogenetic differentiation of *Vigna* species in Asia. in *JIRCAS International Symposium Series no. 2* 112–120 (JIRCAS, MAFF, 1994).

13. Egawa, Y., Nakagawara, M. & Fernandez, G. C. J. Cross compatibility and cytogenetical relationships among Asian *Vigna* species. in *Bruchids and Legumes: Economics, Ecology and Coevolution* 201–208 (Springer Netherlands, 1990). doi:10.1007/978-94-009-2005-7_22

14. Chen, N. C., Baker, L. R. & Honma, S. Interspecific crossability among four species of *Vigna* food legumes. *Euphytica* **32,** 925–937 (1983).

15. Gopinathan, M. C., Babu, C. R. & Shivanna, K. R. Interspecific hybridization between rice bean (*Vigna umbellata*) and its wild relative (*V. minima*): Fertility-sterility relationships. *Euphytica* **35,** 1017–1022 (1986).

16. Fatokun, C. A., Perrino, P. & Ng, N. Q. Wide Crossing in African Vigna Species. in *Advances in Cowpea Research* 50–57 (1997).

17. Fatokun, C. Breeding cowpea for resistance to insect pests: attempted crosses between cowpea and Vigna vexillata. in *Challenges and opportunities for enhancing sustainable cowpea production* 52–61 (2002).

18. Moray, C., Game, E. T. & Maxted, N. Prioritising in situ conservation of crop resources: A case study of African cowpea (*Vigna unguiculata*). *Sci. Rep.* **4,** 5247 (2014).

19. Kang, Y. J. *et al.* Genome sequence of mungbean and insights into evolution within *Vigna* species. *Nat. Commun.* **5,** 5443 (2014).

20. Tun, Y. T. & Yamaguchi, H. Phylogenetic relationship of wild and cultivated *Vigna* (Subgenus *Ceratotropis*, Fabaceae) from Myanmar based on sequence variations in non-coding regions of of trnT-F. *Breed. Sci.* **57,** 271–280 (2007).

21. Vijaykumar, A., Saini, A. & Jawali, N. Molecular characterization of intergenic spacer region of 5S ribosomal RNA genes in subgenus *Vigna*: extensive hybridization among *V. unguiculata* subspecies. *Plant Syst. Evol.* **294,** 39–55 (2011).

22. Pienaar, B. J. & Kok, P. D. F. The *Vigna vexillata* complex (Fabaceae) in southern Africa. *South African J. Bot.* **57,** 236–245 (1991).

23. Pasquet, R. S. & Vanderborght, T. Isozyme polymorphism in the *Vigna frutescens*-*V. membranacea* complex (Tribe Phaseoleae, Fabaceae). *Biochem. Syst. Ecol.* **28,** 29–43 (2000).

24. Pasquet, R. S., Schwedes, S. & Gepts, P. Isozyme diversity in Bambara groundnut. *Crop Sci.* **39,** 1228–1236 (1999).

25. Goel, S., Raina, S. N. & Ogihara, Y. Molecular evolution and phylogenetic implications of internal transcribed spacer sequences of nuclear ribosomal DNA in the *Phaseolus*-*Vigna* complex. *Mol. Phylogenet. Evol.* **22,** 1–19 (2002).

26. Chamberlain, S., Ram, K., Barve, V. & Mcglinn, D. rgbif: Interface to the Global Biodiversity Information Facility. R package version 0.4. 0. (2016).

27. Hijmans, R., Etten, J. van & Cheng, J. Package ‘raster’. (2015).

28. Hijmans, R., Phillips, S., Leathwick, J. & Elith, J. Package ‘dismo’. *Circles* (2016).

29. Pebesma, E., Bivand, R. & Rowlingson, B. Sp: classes and methods for spatial data. (2013).

30. Bivand, R. & Rundel, C. rgeos: Interface to Geometry Engine-Open Source (GEOS). (2012).

31. Bivand, R., Keitt, T. & Rowlingson, B. rgdal: Bindings for the geospatial data abstraction library. *R Packag. version* (2013).

32. Hijmans, R., Williams, E. & Vennes, C. Geosphere: spherical trigonometry. R packag. version 1.3-11. (2014).

33. Bivand, R. & Lewin-Koh, N. maptools: Tools for reading and handling spatial objects. *R Packag. version 0.8–27* (2013).

34. Wickham, H. *ggplot2: elegant graphics for data analysis*. (Springer New York, 2009).

35. Højsgaard, S. The {doBy} package. *R News* **6,** 47–49 (2006).

36. Mendiburu, F. de. Agricolae: statistical procedures for agricultural research. *R Packag. version* (2014). doi:10.1525/california/9780520268326.003.0002

37. Meghwal, R., Joshi, U. & Kumar, S. Screening of moth bean (*Vigna aconitifolia*) core collection against yellow mosaic virus. *Indian J. Agric. Sci.* **85,** 571–575 (2015).

38. Srinives, P., Somta, P. & Somta, C. Genetics and breeding of resistance to bruchids (*Callosobruchus* spp.) in *Vigna* crops : A review. *NU Sci. J.* **4,** 01-17 (2007).

39. Cheema, H. K., Pratap, A. & Sujayanand, G. K. Breeding for insect resistance in mung bean and urd bean. in *Breeding Insect Resistant Crops for Sustainable Agriculture* 353–385 (Springer Singapore, 2017). doi:10.1007/978-981-10-6056-4_12

40. Tomooka, N., Kashiwaba, K., Vaughan, D. A., Ishimoto, M. & Egawa, Y. The effectiveness of evaluating wild species: Searching for sources of resistance to bruchid beetles in the genus *Vigna* subgenus *Ceratotropis*. *Euphytica* **115,** 27–41 (2000).

41. Tomooka, N., Lairungreang, C., Nakeeraks, P., Egawa, Y. & Thavarasook, C. Development of bruchid-resistant mungbean line using wild mungbean germplasm in Thailand. *Plant Breed.* **109,** 60–66 (1992).

42. Pandey, A. K., Burlakoti, R. R., Kenyon, L. & Nair, R. M. Perspectives and challenges for sustainable management of fungal diseases of mungbean [*Vigna radiata* (L.) R. Wilczek var. *radiata*]: A review. *Front. Environ. Sci.* **6,** (2018).

43. Nair, R. M. *et al.* Identification of mungbean lines with tolerance or resistance to yellow mosaic in fields in India where different begomovirus species and different *Bemisia tabaci* cryptic species predominate. *Eur. J. Plant Pathol.* **149,** 349–365 (2017).

44. Togola, A. *et al.* Host plant resistance to insect pests of cowpea (*Vigna unguiculata* L. Walp.): achievements and future prospects. *Euphytica* **213,** 239 (2017).

45. Latunde-Dada, A. O., O’Connell, R. J., Bowyer, P. & Lucas, J. A. Cultivar resistance to anthracnose disease of cowpea (*Vigna unguiculata* (L.) Walp.) caused by *Colletotrichum destructivum* O’Gara. *Eur. J. Plant Pathol.* **105,** 445–451 (1999).

46. Kumar, D., Golakia, B. A. & Parakhia, A. M. Characterization and genetic diversity of cowpea (*Vigna unguiculata* L.) genotypes linked to cowpea yellow mosaic virus. *Legum. Res. An Int. J.* **4,** (2018).

47. Allen, D., Emechebe, A. & Ndimande, B. Identification of resistance in cowpea (*Vigna unguiculata*) to diseases of the African savannas. *Trop. Agric.* (1981).

48. Ng, N. Recent developments in cowpea germplasm collection, conservation, evaluation and research at the Genetic Resources Unit IITA. in *Cowpea genetic resources* (eds. Ng, N.Q. & Monti L.M.) 13–20 (International Institute of Tropical Agriculture (IITA), 1990).
